# Ultrasound as a Method for Early Diagnosis of Breast Pathology

**DOI:** 10.3390/jpm13071156

**Published:** 2023-07-18

**Authors:** Rute Santos, Ana Raquel Ribeiro, Daniela Marques

**Affiliations:** 1 Coimbra Health School, Polytechnic University of Coimbra, 3046-854 Coimbra, Portugal; 2 Laboratory for Applied Health Research (LabinSaúde), 3046-854 Coimbra, Portugal; 3 Radiotherapy Department, Coimbra Hospital and University Center, 3004-561 Coimbra, Portugal; 4 Joaquim Chaves Oncologia, S.A., 2790-225 Carnaxide, Portugal

**Keywords:** ultrasound, breast, breast pathology, ultrasound screening

## Abstract

Introduction: Ultrasound is a non-invasive, low-cost technique that does not use ionising radiation and provides a “real-time” image, and for these reasons, this method is ideal in several situations. Purpose: To demonstrate breast ultrasound evaluation as a first-line diagnostic method and to evaluate the variation of breast characteristics with age. Material and Methods: A total of 105 women with a mean age of 30 years participated and were divided into three age groups: 18–39, 40–59, and 60–79 years, excluding participants subject to mastectomy. After completing the informed consent, all participants answered personal and sociodemographic questions, such as personal and family history, menstrual cycle, pregnancy, ultrasound, and mammography, among others. They were then submitted to a bilateral breast ultrasound examination. Subsequently, all the images and their data were analysed, and a technical report of the examination was given to all the participants. Results: A total of 105 women with a mean age of 30 years participated, 58 of whom underwent the examination for the first time. In 31, changes (of which only 7 were known) were diagnosed. It was verified that, according to age group, the density of the breast stroma varied; older women have less breast density. Conclusions: Ultrasound is a good method for breast evaluation and can be considered important for the early evaluation of breast pathology and follow-up of the pathology.

## 1. Introduction

The breast is composed of Cooper’s ligaments (fibrous bands that represent the natural support of the breast). This is the most prominent surface structure on the anterior chest wall, located between the second and sixth ribs on the vertical axis and between the outer edge of the sternum and the midaxillary line on the horizontal axis (in an adult) and can be divided into four quadrants—the upper outer quadrant, the lower outer quadrant, the upper inner quadrant, and the and lower inner quadrant—to make it easier to locate and describe tumours [1,2,3]. The mammary glands are found in the subcutaneous tissue covering the pectoral muscles.

Breast size is determined by the amount of fat surrounding the glandular tissue. Glands are composed of lobules interspersed with glandular, connective, and adipose tissue. Lobes are subdivided into lobules composed of alveoli. The lobes connect to the nipple through the galactophore ducts. The nipple is surrounded by the areola [1,2,3]. Arterial irrigation is performed by the branches of the internal thoracic artery, the branches of the axillary artery, and the posterior intercostal arteries[1,2,3]. Despite the existence of some drainage to the internal thoracic vein, the venous drainage of the breast is principally to the axillary vein. The lymphatic drainage of the breast is important due to its role in cancer cell metastasis [3]. The lymphatic drainage of the breast is important due to its role in cancer cell metastasis [2,3,4]. From the mammary papilla, areola, and gland lobules, the lymph flows to the subareolar lymphatic plexus, and then, most of this lymph, especially the lymph coming from the lateral breast quadrants, drains to the axillary lymph nodes. The remainder of the lymph, coming from the medial quadrants, drains to the parasternal lymph nodes or to the opposite breast [1,3,4]. Lymphatic vessels in the breast skin, except in the breast papilla and areola, drain the axillary, lower deep cervical, and infraclavicular lymph nodes and the parasternal lymph nodes on both sides [1,3,4]. The lymph from the axillary lymph nodes is drained to the infraclavicular and supraclavicular lymph nodes, and from these, it passes to the subclavian lymphatic trunk, which also drains lymph from the upper limbs. The lymph from the parasternal lymph nodes enters the bronchomediastinal trunk, which drains lymph from the thoracic viscera [1,3,4].

Nowadays, there is a higher incidence of breast pathologies not only due to the advancement of technology that allows the early detection of pathologies but also due to the awareness of society at this level. Over time, norms have been created to try to change the panorama of the increase in pathologies associated with the breasts, with the emergence of screening tests: mammography (usually for those over 50 years of age), ultrasound (a complementary method to mammography), and physical examination (breast self-palpation). The last one is extremely important for the woman to know her body [5,6]. Other imaging tests, such as mammography and breast ultrasonography, are usually performed first, but MRI might be performed if the results of these tests are not clear. Although not recommended as a screening method in the general population, MRI should be performed in women at high risk of breast cancer due to family history or BRCA1/2+ [5,7]. Breast MRI is a sensitive modality for the detection of breast cancer. However, cases of false negatives may occur, where the cancer is not visualised on MRI and is diagnosed with another imaging modality. Careful development of thorough search patterns is critical to avoid these errors. Cognitive errors occur when an abnormality is identified but misinterpreted or mischaracterised as benign. Cases of false negatives are inevitable, as certain subtypes of breast cancer, including ductal carcinoma in situ, invasive lobular carcinoma, and certain well-differentiated invasive cancers, may demonstrate little or no enhancement on MRI due to differences in angiogenesis and neovascularisation. MRI is a valuable diagnostic tool in breast imaging. However, MRI should continue to be used as a complementary modality, together with mammography and US, in the detection of breast cancer [8].

Ultrasonography is a non-invasive imaging technique and may be repeated as often as necessary since it is painless, inexpensive, and does not involve exposure to ionising radiation [6,9,10].

Although mammography can detect breast pathology and thus reduce breast cancer mortality, it has been described in the literature as an imperfect tool that is not as effective, particularly in the female population with dense mammary glands [11,12,13].

Breast ultrasound is a more sensitive and accurate modality than mammography for the early diagnosis of breast cancer in Chinese women with suspected breast lesions. This might also be true for all women with dense breasts worldwide. For older or obese women, mammography can be used to supplement ultrasound to increase sensitivity, specificity, and accuracy. However, long-term follow-up is still needed to assess whether ultrasound reduces breast cancer mortality [14].

Ultrasonography performs real-time detection of breast lesions and evaluates their morphological characteristics, such as shape, echogenicity, nodules, and cysts [15].

The breast is composed of lobules interspersed with adipose tissue and connective tissue. These fat layers are heterogeneous and hypoechogenic. The stroma of the breast is much more hyperechogenic. Fat lobules, contained within the breast stroma, can be mistaken for masses. Planar sheets composed of fibrous connective tissue provide structure to the breast. Cooper’s ligaments are thin linear echogenic bands that ascend from the chest wall. The lactiferous ducts are distinct hypoechogenic tubular structures radiating from the nipple and should be a maximum of 2 mm in diameter (identified under the areola), and, subsequently, they drain into the lactiferous sinus (maximum 4 mm) [16,17].

Posterior to the breast, the pectoral muscles can be seen as a slightly hypoechogenic area with linear strands. The ribs are posterior to this musculature and have attenuating properties that cause artefacts. Other areas that cause similar artefacts are the nipple area and the areolar region. Compression is vital in differentiating a mass from the attenuating anatomy [16].

Lymph nodes are usually found in the breast, but they are more predominant in the axillary region, are frequently isoechoic with the surrounding breast parenchyma, and may be difficult to identify [16]. On the other hand, there are several factors that are thought to influence the existence or increase the incidence of breast pathology. Alcohol consumption, smoking, and obesity, for example, are factors that are believed to influence the predisposition to breast carcinoma [18,19]. Many of the compounds that result from smoking are considered carcinogenic, namely polyaromatic hydrocarbons (PAHs). These are associated with the development of various types of cancer and may participate in tumour progression, as they show an inducing effect on proliferation in various cancer cell lines [20,21]. Regular alcohol consumption is also a risk factor for breast pathologies, acting as a co-carcinogen and increasing the permeability of cell membranes to carcinogens [22].

Pregnancy, due to the influence of prolactin on the growth of ductal, lobular, and alveolar structures in the last half of pregnancy, has a protective effect against the development of breast cancer. On the other hand, multiparous women who have their first child at an advanced age present a twofold higher risk of developing breast cancer [18,23].

Among the significant risk factors for the development of a second breast cancer, personal history of breast cancer stands out [24,25].

Women with a history of endometrial, ovarian, and colon cancer are also at an increased risk of developing breast cancer compared with the general population [18,24]. The risk of developing breast cancer also increases if there is a family history of this pathology, especially at younger ages (>40/45 years). In addition, having other family members with this pathology on the maternal or paternal side of the family can also increase risk. The sharing of genetic characteristics is more likely among first-degree relatives (mothers, fathers, brothers, sisters, sons, and daughters) [18,19,20,21,22,23,24,25,26]. Family history is, however, a heterogeneous risk factor that depends on the number of relatives with the same pathology, the age at diagnosis, and the number of affected relatives [18,19,20,21,22,23,24]. Menstruation is also very important and has an influence on the evaluation of breast pathologies by ultrasound. Depending on which phase of the menstrual cycle a participant is in, the breast stroma can become dense, which may give rise to false positives. The follicular phase of the menstrual cycle is the best time to take the test [18,27]. Recently, it has been observed that the use of oral contraceptives is associated with many non-contraception-related benefits, one of which is a decrease in the risk of benign breast alterations [18,28].

According to one study, women with dense breasts are more predisposed to breast cancer, on the other hand, mammography in these women is less sensitive.; the addition of ultrasound screening can increase breast cancer detection rates by 1.9–4.2%, depending on the population [12]. Automated ultrasound devices can mitigate the challenges posed by portable screening programmes; notably, they allow faster examination times, reduced operator dependence, and improved workflow and datasets. However, automated screening ultrasound has barriers to its implementation, including the need for additional training, the cost of the device, and possible integration into pre-existing PACS. Continued experience with this modality, however, demonstrates an acceptable collection rate and sensitivity and results in better detection rates for clinically important cancers [29].

Breast cancer is the most frequently diagnosed pathology in women, and it is the second cause of death from cancer, and it represents one-third of all cancer cases. However, due to increasingly earlier detection associated with increased screening, awareness, and adherence, as well as better therapeutic strategies, the number of cases of breast cancer has been decreasing [30].

Breast cancer incidence does not show a uniform geographic distribution. The incidence rate is higher in developed countries, but it has been shown to be increasingly prevalent in developing countries [31].

In Portugal, breast cancer is the most frequently diagnosed malignant neoplastic pathology in the female population and is considered the second leading cause of death in women [32]. In 2020, around 7000 new cases of breast cancer were detected, leading to the deaths of around 1800 patients [33].

That said, it would be beneficial if the word “early” was associated with breast ultrasound. Ultrasound has the potential to yield high sensitivity and specificity in the detection of breast cancer. Ultrasound is widely available, easy to maintain, inexpensive, durable, and easily transportable. In view of the increasing global burden of breast cancer and the lack of access to timely detection through imaging, ultrasound can be an effective primary detection tool and screening method for breast lesions, particularly in resource-poor settings where mammography is not available [34].

Despite the enormous technological and scientific evolution in the last few years in the approach to breast cancer diagnosis and treatment, organised population screening remains a controversial topic, and there is no international consensus on the best way to implement it. Future research will involve the study and implementation of patient-centred models based on each patient’s clinical and genetic characteristics, allowing the definition of individualised recommendations for when to start, at what age to suspend, and how often to perform screening according to the individual risk determined; this will allow for better risk stratification and risk–benefit balance associated with breast cancer screening, which, ideally, will lead to increased early detection of breast cancer, a reduced mortality risk, and reduced rates of false positives and overdiagnosis [35].

It is known that younger people are more easily predisposed to undergo this examination instead of mammography. On the other hand, by detecting the pathology earlier, that is, by detecting breast alterations at an early stage, the prognosis may be more favourable.

Given the above, this study primarily aimed to highlight the early assessment of breast pathology by ultrasound and its importance. The secondary aim was to evaluate breast characteristics throughout the course of ageing.

Also, this study aim was to confirm ultrasound as a screening test to raise awareness of its importance and the importance of breast assessment.

## 2. Materials and Methods

### 2.1. Participants

In this study, 105 female participants aged between 18 and 79 years were evaluated. This sample was divided into three groups according to the following age division: 18 to 39 years old; 40 to 59 years old; and 60 to 79 years old.

The division was fair considering the decrease in stromal density with increasing age in female patients and according to the variables available in the sociodemographic questionnaire regarding the participant’s general and personal information, such as family history, age, menopause, taking the pill, and pregnancy, among other factors.

In the present study, participants who had already undergone mastectomy were not accepted. The entire procedure for data collection was explained in advance, and after answering a sociodemographic questionnaire, the participants signed an informed consent form. This study was approved by the Ethics Committee of the Polytechnic of Coimbra (No. 5/2018).

### 2.2. Procedures

To carry out this study, the acquisition protocol followed the European guidelines [36] with the aim of reproducing the acquisition of images in the entire sample.

Each participant was given a questionnaire assessing several essential details, such as age, smoking habits, alcohol consumption, menstrual cycle, pregnancy, and personal and family history, among others.

During the breast ultrasound, the participant was positioned supine with a slight inclination to the contralateral side.

Scanning was performed clockwise, and a total of 12 images (minimum) were acquired after bilateral evaluation. General Electric Healthcare’s Logiq E ultrasound equipment (GE Ultraschall, Deutschland) and a 7–12 MHz linear probe was used.

For better acoustic contact without causing too much pressure on the participants’ breasts, a water-based gel was used. In this way, it was possible to obtain a good characterisation and visualisation of the breast stroma.

All images were acquired by students pursuing a degree in medical imaging and radiotherapy with the supervision and guidance of an experienced professional in the field of breast ultrasound.

In this study, the Pearson correlation test was used with the aid of the IBM SPSS Statistics Version 22.0 software to measure the degree of association between two variables). The values can be interpreted as follows (by convention) [33]:

- r < 0.2: very low linear correlation;
- 0.2 < r < 0.39: low linear correlation;
- 0.4 < r < 0.69: moderate linear correlation;
- 0.70 < r < 0.89: high linear correlation;
- 0.9 < r < 1: very high correlation.

## 3. Results

Of the 105 participants, 30% (31 cases) had changes in the breast stroma, and the remaining 70% did not have any type of changes.

**Figure 1:**
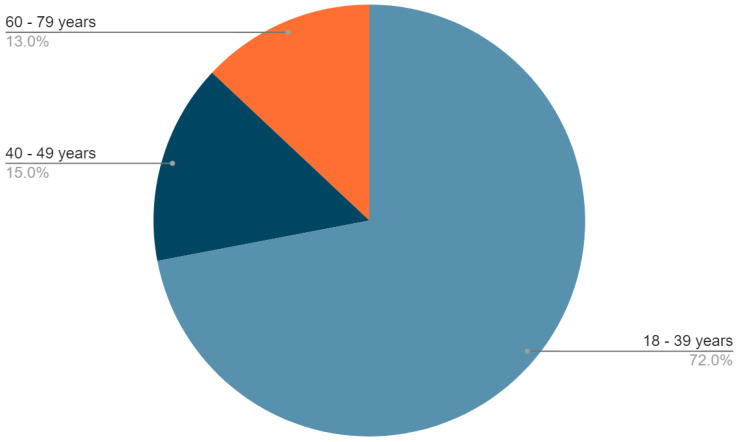
Percentage of participants in each group, according to age.

Of the participants who had alterations (31 cases), only 21% (7 cases) were aware of them. Initially, participants were divided into three groups by age: 72% of participants were 18 to 39 years old, 15% were 40 to 59 years old, and 13% were 60 to 79 years old (Figure 1).

Of the total number of participants surveyed, 33.4% responded that they had a family history of breast pathology, and 11.4% responded that they already had a personal history.

When asked if they performed breast self-exams, 53.3% of the participants admitted that they did not.

Only 30% of the study participants already had children. Of the 84% of menstruating participants, 61% used a contraceptive method.

Regarding the consumption of alcohol, most participants denied drinking alcohol frequently (97%), and only 16% had a smoking habit.

**Figure 2:**
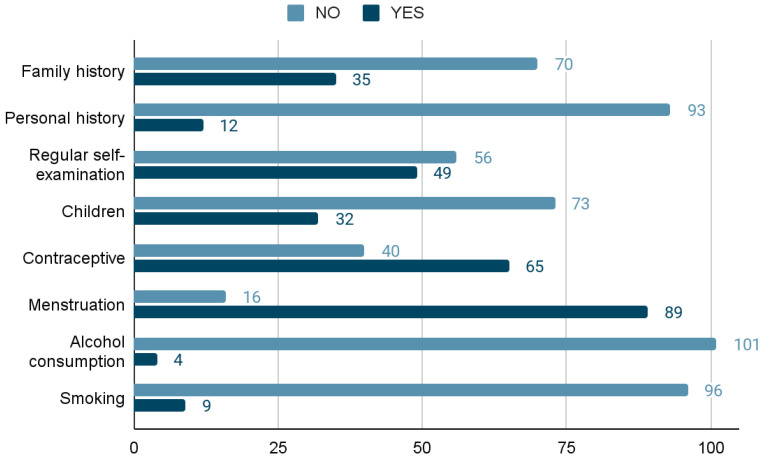
Graph representing the answers given by the participants to the sociodemographic questionnaire.

All responses to the sociodemographic questionnaire are shown in Figure 2.

When analysing the presence or absence of alterations in the breast stroma by age group, it was possible to notice that among the participants of group 1 (18–39 years old), 28.9% had breast alterations; among those in group 2 (40–59 years old), 37.5% had breast lesions; and finally, among those in group 3 (60–79 years), 23.1% had changes (Table 1).

Of the total number of participants with breast alterations, lesions on the left (20.2%) were more common than on the right (17.2%). After three months, 18 of the participants who had breast changes were contacted to repeat the exam and find out which of the changes (if any) remained. Of these participants, nine maintained the changes, three cases were proven to be false positives (cycle phase or trichotomy), and the remaining six women did not repeat due to schedule incompatibilities.

The correlation between the variables was analysed, with the presence/absence of breast alterations presenting a positive correlation with personal history (*p* < 0.05) and age presenting a positive correlation with other exams that the participants had already performed (*p* < 0.05). None of the other variables in this study were significantly correlated according to Pearson’s test.

However, the correlations of these variables were low (r between 0.2 and 0.39—low linear correlation) when the variables were the presence/absence of breast alterations and personal history, and they were moderate for age and other exams that the participants previously performed (r between 0.4 and 0.69—moderate linear correlation).

## 4. Discussion

In this study, it was possible to verify that breast alterations can occur in any age group and that there are several external factors that can act as risk or safeguard factors. Therefore, it is important to use breast ultrasound from an early age and not from a certain age.

Currently, the first-line test for early diagnosis of breast pathology is mammography. However, it is an imperfect tool that is not equally effective for all women. Overall, the sensitivity of mammography for detecting breast cancer is 85%. However, in women with dense breast tissue, the sensitivity of mammography is lower (47.8–64.4%) [12].

Although breast density tends to decrease with age, it is a significant problem in women of all ages; as breast density increases, so does the chance of developing breast cancer [13,37,38].

Ultrasound of the breast may be a solution for detecting breast cancer in women with dense breast tissue for whom mammography is less effective and in women who have an increased risk of breast cancer. Women with dense breast tissue constitute the largest group of intermediate-risk women for whom mammography may not be sufficient. Breast ultrasound detects small lesions that are clinically significant, invasive, and predominantly present with negative lymph nodes [13] that are not detected in mammography.

Analysing the obtained data in this study, it is possible to acknowledge that in group 2 (40–59 years), there was a higher incidence of breast alterations. However, it was also observed that in group 1 (18–39 years), the percentage of alterations was high. The incidence of breast lumps was relatively higher in childbearing-age women [13,39,40].

It was also confirmed that as participants’ ages increased, the density of the breast stroma decreased [41,42].

The positive correlation between the presence or absence of breast alterations and personal history (*p* < 0.05) suggests that with their increase, the incidence of breast alterations increases as well.

Regarding the remaining variables, no significant relationships were found. This may be because the sample was small or because there were some inconsistencies in the collection of data through the questionnaires.

A study by [43] observed a natural chronology of the disease associated with age [32].

It should be noted that US does not replace mammography, but they can complement each other [11,32,37,44]. In a study by [13], the sensitivity of mammography combined with ultrasound was greater than that of mammography alone (76% vs. 52%), with a decrease in specificity from 91% with mammography to 84% with mammography combined with ultrasound [37].

The overall sensitivity of mammography only was 61.5% in women with dense breasts and 86.6% in women with non-dense breasts. The sensitivity of mammography plus ultrasound was 81.3% in women with dense breasts and 95.0% in women with non-dense breasts [37].

Breast self-examination can also provide important information for an early diagnosis, and if the nodule is more superficial, it is more easily detected.

Women who regularly perform breast self-examinations may note changes in the breasts during their cycle. The breasts become lumpier and tender before the menses and less lumpy and less tender after the menses [43,45].

In this study, of the 56 participants who did not undergo regular breast palpation, 17 had breast changes, and self-examination could have been an important tool to detect this change earlier. No malignant changes were detected throughout the study.

Ultrasound plays a more significant role in differentiating between cysts and solid masses [13,39,46].

Despite these results, the study has some limitations, namely, the average age and the number of participants, which did not allow the sample to be organised into smaller groups.

## 5. Conclusions

In this study, it was proven that evaluation by ultrasound has numerous advantages as a complement or when combined with mammography and physical examination insofar as it helps to diagnose breast pathology at an early stage. Through this technique, benign alterations (nodules and cysts) were detected in the participants.

It was thus concluded that it is extremely important to breast tissue changes early so that there can be a follow-up of the evolution of the alterations, which could have several advantages.

The results were quite positive, as the female population generally participated freely, and there were only difficulties in the second evaluation phase due to the incompatibility of schedules and the fact that it was necessary to wait 3 months after the first evaluation.

The present study found that the participants did not carry out breast self-examination as often as would be desirable, as this is the first step towards the early detection of any pathology associated with the breast.

Finally, it was concluded that evaluation by breast ultrasound should be easily accessible because, in addition to its advantages, it is a technique more easily accepted by the population due to its painlessness.

Therefore, the female population will be able to know their bodies better and achieve early diagnosis of malignant breast pathology at an early stage, consequently increasing the success of treatment and reducing the mortality rate due to breast cancer.

## Figures and Tables

**Table 1:** Presence of breast alterations according to age.

Groups				
		1 (18–39)	2 (40–59)	3 (60–79)
Breast Alterations	Yes	22–28.9%	6–37.5%	3–23.1%
	No	54–71.1%	10–62.5%	10–76.9%
Total		76–100%	16–100%	13–100%
